# Renewable bio-based adhesive fabricated from a novel biopolymer and soy protein

**DOI:** 10.1039/d1ra00766a

**Published:** 2021-03-23

**Authors:** Shiqing Chen, Yuan Chen, Zongtao Wang, Huan Chen, Dongbin Fan

**Affiliations:** Research Institute of Wood Industry, Chinese Academy of Forestry Beijing 100091 China fandongbin8@163.com +86-10-62881937 +86-18500236090

## Abstract

In this study, a bio-based soy protein adhesive derived from environmentally friendly and renewable enzymatic hydrolysis lignin (EHL), epoxidized soybean oil (ESO), and soy protein isolate (SPI), was successfully prepared. A novel biopolymer (EHL-ESO), as a multifunctional crosslinker, was firstly synthesized from modified EHL and ESO, and then crosslinked with soy protein isolate to obtain a bio-based soy protein adhesive. The structure, thermal properties, and adhesion performance of the obtained soy protein adhesives were determined by Fourier transform infrared spectroscopy (FTIR), thermogravimetric analysis (TGA), scanning electron microscopy (SEM) and wet shear strength. The maximum degradation temperature of SPI/EHL-ESO adhesives (332–343 °C) was higher than that of the pristine SPI adhesive (302 °C). Moreover, plywood bonded by the modified adhesive reached a maximum wet shear strength value of 1.07 MPa, a significant increase of 101.8% from the plywood bonded by pristine SPI adhesive. The enhancements in the thermal stability and wet shear strength were attributed to the formation of a dense crosslinking network structure. This work not only highlights the potential to replace petroleum-based polymers, but also presents a green approach to fabricate fully bio-based soy protein adhesive for preparing all-biomass wood composite materials.

## Abstract 2

The crosslinking reaction process of SPI/EHL-ESO adhesives.

## Graphical abstract

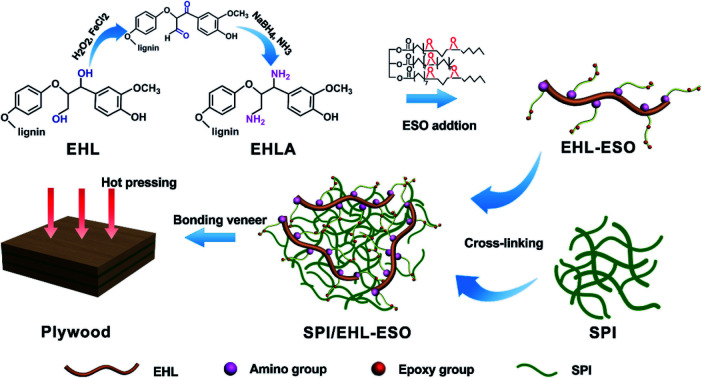

## 1. Introduction

In recent decades, interest in developing environmentally friendly and renewable adhesives has grown in order to offer alternatives to formaldehyde-based resins, including phenol formaldehyde (PF)-based and urea formaldehyde (UF)-based resins, as they are derived from non-renewable petrochemicals. Moreover, the resulting composite wood panels release formaldehyde, which is a carcinogen that negatively effects human health and the environment.^1^ Thus, the use of renewable biomasses as biodegradable adhesive products has received considerable research attention. Soy protein isolate (SPI) is a by-product of the soybean oil industry and an ideal renewable raw material for manufacturing wood adhesives, as it is inexpensive and highly abundant, with good biocompatibility and biodegradability.^2–4^ However, its poor mechanical properties and low water resistance are the main problems that hinder the use of soy protein-based materials.

Recently, a series of chemical modifications have been reported to enhance the performance of soy protein adhesives, such as soy protein structural and crosslinking agent modifications.^5^ Specifically, epoxy and melamine resin, polyisocyanate, polyamidoamine-epichlorohydrin (PAE) resin, phenol formaldehyde resin, trimethylolpropane triglycidyl ether (THPTG), and bisphenol-A waterborne epoxy resin and polyacrylamide (PAM),^6–11^ were applied to enhance the water resistance of soy protein adhesive due to the crosslinked network structure formed by the reactions with the hydrophilic groups (*i.e.*, –NH_2_, –COOH, and –OH) in soy protein molecules. The resulting adhesives meet the interior use requirements but still rely on unrenewable petroleum-based resources.

To ensure the renewability and sustainability of the soy protein adhesive, natural polymers, such as lignin, tannin, and fiber, have been applied to increase water resistance of the soy protein adhesive.^5,12,13,18,21^ Lignin, as the second most abundant organic biopolymer, has attracted great attention due to its both phenolic and aliphatic hydroxyl groups that can be converted to other functional groups, providing the possibility of producing value-added products.^18,32^ It has been demonstrated that low molecular weight lignin mixing with soy protein increased the adhesive strength of soy protein-based materials.^12^ In addition, the addition of sorghum lignin (SL) and extruded SL to SPIs or modified soy protein has been shown to increase the shear strength and water resistance.^13^ However, mere hydrogen bonding interactions are usually not enough to ensure sufficient mixing of lignin and protein.^12–14^ Therefore, it is necessary to enhance the compatibility of lignin with soy protein. Chemical modification, which increases the intermolecular covalent bonds, can be regarded as a feasible strategy to improve the reactivity of lignin, including amination, epoxidation, and phenolation.^15,16,18^ Xin *et al.* reported that lignin *via* Fenton oxidation and reductive amination reaction introduced amino groups, which can achieve better dry and wet shear strengths of modified soy protein adhesive.^18^ However, the mechanism of the reactions between lignin amine and soy protein molecules was not revealed. In addition, the improvement of lignin reactivity by only amination modification is limited, thereby failing to significantly improve the water resistance of modified soy protein-based adhesive.

Epoxidized soybean oil (ESO) can be used to further improve the reactivity of modified lignin and to achieve multiply crosslink with soy protein molecules, as it has at least 5 epoxy groups per molecule. ESO is a nontoxic, low cost, and renewable material often used as plasticizer, stabilizer, or reactivity modifier.^19^ The soy protein film prepared by crosslinking with ESO demonstrated improved water resistance and tensile strength.^20^ Zhao *et al.* grafted ESO with phosphorus groups and then crosslinked with kenaf fiber to produce high performance and water-resistant biomass soy protein adhesive, because ESO can enhance interactions between soy protein and fiber.^21^ Thus, a multifunctional and reactive biopolymer that is synthesized from lignin and ESO, is expected to be a sustainable and viable strategy for soy protein adhesives modification.

The aims of this work were the improvement of the lignin reactivity and development of a high-performance (*i.e.*, water and thermal resistant) soy protein-based adhesive. A novel biopolymer (EHL-ESO) as a multifunctional crosslinker (EHL-ESO) with high reactivity was first prepared using lignin and ESO, and then applied to modify soy protein to obtain soy protein-based adhesives. The physicochemical properties of EHL-ESO biopolymer were characterized with Fourier transform infrared spectroscopy (FTIR), proton nuclear magnetic resonance spectrometry (^1^H-NMR), gel permeation chromatography (GPC), and thermogravimetric analysis (TGA). The effects of EHL-ESO biopolymer on water resistance, mechanical and thermal properties of the soy protein-based adhesive were also studied.

## 2. Materials and methods

### 2.1 Materials

SPI (>95% protein content) was provided by Hebei Baiwei Biological Technology Co., Ltd. (Hebei, China). The enzymatic hydrolysis lignin (EHL) was supplied by Jinzhou Lingyu Chemical Co., Ltd. (Liaoning, China). ESO and sodium borohydride (NaBH_4_, 98%) was provided by Shanghai Aladdin Bio-Chem Technology Co., Ltd. (Liaoning, China). Iron(ii) chloride tetrahydrate (FeCl_2_·4H_2_O) was provided by Beijing Kangpu Huiwei Technology Co., Ltd. (Beijing, China). Hydrogen peroxide (30.0%) and sodium hydroxide (NaOH) were provided by Beijing Chemical Works (Beijing, China). Ammonia solution (25% aq.) was obtained from Tianjin Huadong Reagent Factory (Tianjin, China). Anhydrous methanol (CH_3_OH) was provided by Beijing Huateng Chemical Co., Ltd. (Beijing, China). Poplar wood veneer (300 mm × 300 mm × 1.7 mm, 10–12% of moisture content) was provided by Wen'an (Hebei, China).

### 2.2 Purification of EHL

The crude EHL was firstly treated with a 2% aqueous sodium hydroxide solution to remove impurities. And then the suspension was centrifuged and filtered. The product was precipitated by using the hydrochloric acid solution to adjust the filtrate pH to 2.0. Finally, the precipitated product was centrifuged, washed with hydrochloric acid solution (pH = 2.0), and freeze-dried to obtain the purified EHL.

### 2.3 Preparation of EHLA

EHLA was obtained as previously reported using Fenton oxidation and reductive amination,^18^ but made some minor changes. Specifically, EHL (11.0 g) and deionized water (110 mL) was added into a three-necked flask and vigorously stirred under ambient conditions for 10 min. FeCl_2_·4H_2_O (1.06 g) was added and stirred for another 20 min. 10 mL of an aqueous hydrogen peroxide solution was then added to this solution drop by drop, and the obtained solution was continuously reacted at 40 °C for 5 h. The oxidized lignin was collected by filtration and washed with deionized water, and then freeze-dried.

Oxidized lignin (5.68 g) and ammonia (12.11 mL) was dissolved in methanol (160.05 mL) stirred under ambient conditions for 20 min. NaBH_4_ (2.4 g) was then added slowly and continuously reacted at 55 °C for 2 h. The resulting mixture was then quenched with H_2_O (30 mL). The mixture was first concentrated by removing the solvent and then washed with deionized water. The enzymatic hydrolysis lignin amine (EHLA) was collected after freeze-drying.

### 2.4 Synthesis of EHL-ESO

To synthesize the EHL-ESO biopolymer as a multifunctional crosslinker, EHLA (3.5 g) and a 1% aqueous sodium hydroxide solution (140 g) was placed in a three-necked flask, stirring at 40 °C for 20 min. ESO (35 g) was then added and continuously stirred at 40 °C for 3 h, and finally EHL-ESO was obtained.

### 2.5 Preparation of soy protein-based adhesives and application in plywood bonding

The deionized water (88 g) was used to dissolve SPI (12 g) at ambient conditions and the mixture was stirred until forming a homogeneous system *i.e.*, SPI adhesive. SPI/EHLA adhesive was produced by stirring a solution of EHLA (0.3 g) in 87.7 g of deionized water, then adding SPI (12 g). SPI (12 g) was mixed with ESO (2.7 g) in 85.3 g of deionized water to prepare SPI/ESO adhesive. Finally, SPI/EHL-ESO adhesive system was fabricated by dissolving mixtures of various amounts of EHL-ESO (14, 23, 32, or 42 g) in deionized water (74, 65, 56, or 46 g, respectively). SPI (12 g) was then added, and the solution was stirred to form a homogeneous system (SPI/EHL-ESO-*n*), in which *n* represents the weight percent of EHL-ESO in the adhesives (*n* = 3, 5, 7, or 9). Table 1 shows the formulations for the mentioned adhesives.

**Table 1:** The formulations of different soy protein-based adhesives

Samples	SPI (g)	DW (g)	EHLA (g)	ESO (g)	EHL-ESO (g)
1 SPI	12	88	—	—	—
2 SPI/EHLA	12	87.7	0.3	—	—
3 SPI/ESO	12	85.3	—	2.7	—
4 SPI/EHL-ESO-3	12	74	—	—	14
5 SPI/EHL-ESO-5	12	65	—	—	23
6 SPI/EHL-ESO-7	12	56	—	—	32
7 SPI/EHL-ESO-9	12	46	—	—	42

The manufacturing conditions of the three-layer plywood were as follow: coating density (per surface) of 200 g m^−2^, hot-pressed temperature of 130 °C, and pressure of 1.0 MPa, time of 6 min. The panels were stored under ambient temperature for 24 h after hot pressing.

### 2.6 Characterization

FTIR analysis was conducted on a Nicolet 7600 spectrometer (Nicolet Instrument Corporation, Madison, WI) at wavenumber ranging from 4000 to 400 cm^−1^. Each sample was scanned 32 times, with a resolution of 4 cm^−1^. KBr was previously dried and then the background spectra was collected before testing.^22^ Prior to testing, EHL-ESO was washed using ether and freeze-dried.

EHL and EHLA (20 mg), ESO (20 mg), and EHL-ESO (25 mg) were dissolved in DMSO-d_6_ (1 mL), CDCL_3_ (1 mL), and D_2_O (1 mL), respectively.^23^ EHL-ESO was treated with ether and freeze-dried, and EHL and EHLA were acetylated prior to testing. The ^1^H NMR spectrum of samples was tested using a 400 MHz AVANCE III Bruker NMR spectrometer.

The thermal properties of EHL, EHLA, EHL-ESO and cured soy protein-based adhesives were run on a TG209F1 Libra (NETZSCH, Germany). Prior to testing, EHL-ESO was treated with ether and freeze-dried. Each sample of 10–20 mg was weighed and scanned in the 25–600 °C range at a heating rate of 10 °C min^−1^ under nitrogen flow of 40 mL min^−1^.

The molecular weights of EHLA and EHL-ESO were determined by GPC analysis. Injections were performed using a US Rheodyne7725i syringe with a 20 μL loop. The column was an aqueous phase gel column (molecular weight 300–50 W). A 0.1 N NaNO_3_ + 0.06% NaN_3_ aqueous solution was used as the mobile phase at a flow rate of 0.6 mL min^−1^.

The cross-sectional morphologies of SPI and SPI/EHL-ESO adhesives were conducted on a scanning electron microscopy (SEM, Hitachi S-3400N).

The shear strength of plywood panels (Type II) was measured according to the procedure described in the China National Standards (GB/T 9846-2015). Specifically, 18 plywood samples (25 mm × 100 mm) were obtained from the resulting panels and immersed in water for 3 h at 63 ± 2 °C. Prior to evaluating, the specimens were cooled at ambient temperature approximately 10 min.

## 3. Results and discussion

### 3.1 Characterization of EHL-ESO

#### 3.1.1 Chemical structure analysis

**Fig. 1:**
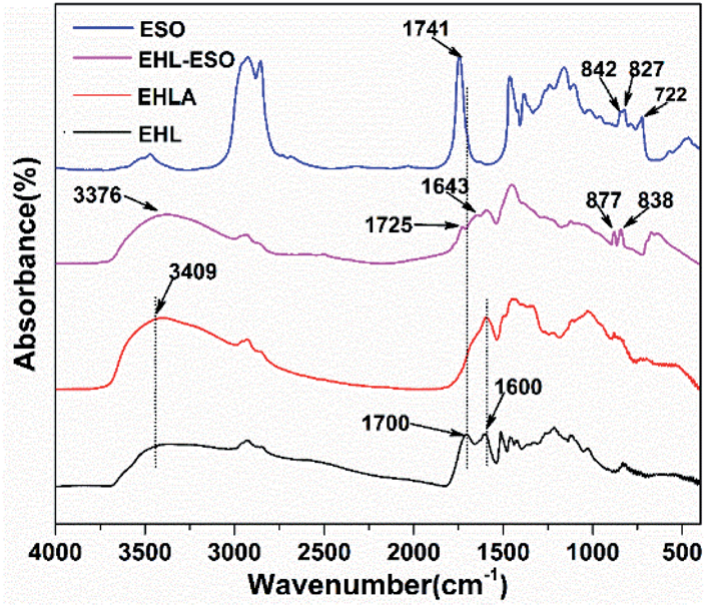
FTIR spectra of EHL, EHLA, ESO, and EHL-ESO.

EHLA was conducted to increase its water solubility and reactivity with nucleophiles, enabling the epoxy groups in ESO reacted with amino groups in EHLA to form a multifunctional crosslinker (EHL-ESO). FTIR spectroscopy was applied to characterize the chemical structure of EHLA and EHL-ESO to confirm ESO grafted successfully. As shown in Fig. 1, the peaks at 1700–1741 cm^−1^ and 1027 cm^−1^ was corresponded to the C=O vibrations (COOH or COOR) and C–O vibrations, respectively.^25^ A broad band at 3409 cm^−1^ of EHL belonged to the free and bound NH– and O–H bending vibrations. Compared to EHL, a peak at 1700 cm^−1^ disappeared in the EHLA, which was due to the carbonyl of the oxidized lignin formed a hemiaminal species that subsequently lost one molecular of water to form an imine intermediate, and then the intermediate was reduced by NaBH_4_ to obtain EHLA.^18^ In addition, the peaks intensities at 1600 cm^−1^ (–NH_2_ deformation) and 3409 cm^−1^ (N–H stretching) increased obviously. These results indicated that the majority hydroxyl groups in EHL were converted to amino groups through Fenton oxidation and reductive amination.

After grafting ESO onto EHLA to form EHL-ESO, the peaks at 1600, 1741, and 3409 cm^−1^ shifted to the lower wavenumbers at 1566, 1725, and 3376 cm^−1^, respectively, indicating that ESO was successfully introduced onto EHLA. Moreover, a new peak appeared at 1643 cm^−1^, corresponding to the bending vibration of N-H,^27^ and a peak at 722 cm^−1^ disappeared, belonging to the bending (rocking) of –(CH_2_)*n*– and –HC=CH–.^26^ Peak intensities at 825 and 843 cm^−1^ (epoxy group)^26^ decreased and shifted to higher wavenumbers at 838 and 877 cm^−1^, respectively, indicating that a ring-opening reaction occurred between amino groups (EHLA) and epoxy groups (ESO). In summary, EHL-ESO was successfully prepared.

**Fig. 2:**
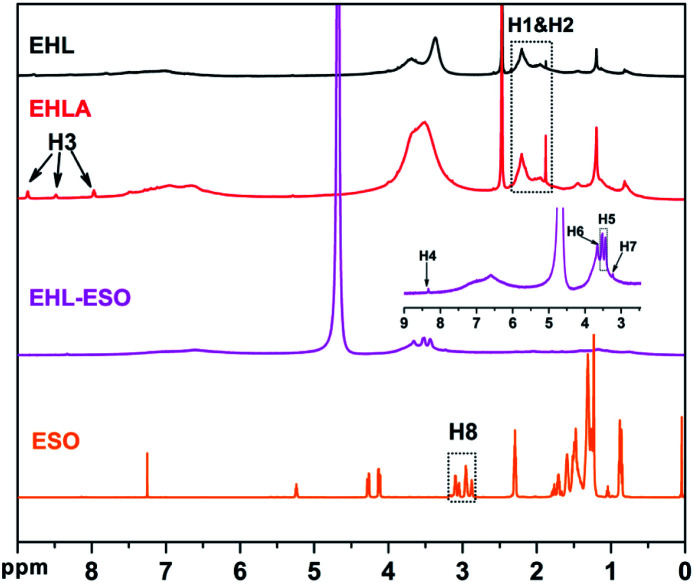
^1^H NMR spectra of EHL, EHLA, ESO, and EHL-ESO.

^1^H NMR spectroscopy was used to further confirm the formation of EHLA and EHL-ESO, as shown in Fig. 2. The ^1^H NMR spectra of EHL showed the peaks at 1.8–2.05 and 2.08–2.35 ppm (H1&H2), which was attributed to acetoxy group protons (CH_3_C=O) in aliphatic and aromatic acetates, respectively.^24,28^ After the Fenton oxidation and reductive amination of EHL, three new signals appeared at 8.86, 8.49, and 7.97 ppm (H3), which was assigned to the protons of –NH– in different acetamide (RNHOCCH_3_) units that formed in the acetylation of the amino groups in EHLA.^18^ This indicated that EHLA was successfully produced *via* Fenton oxidation followed by reductive amination, which was consistent with above FTIR results.

**Fig. 3:**
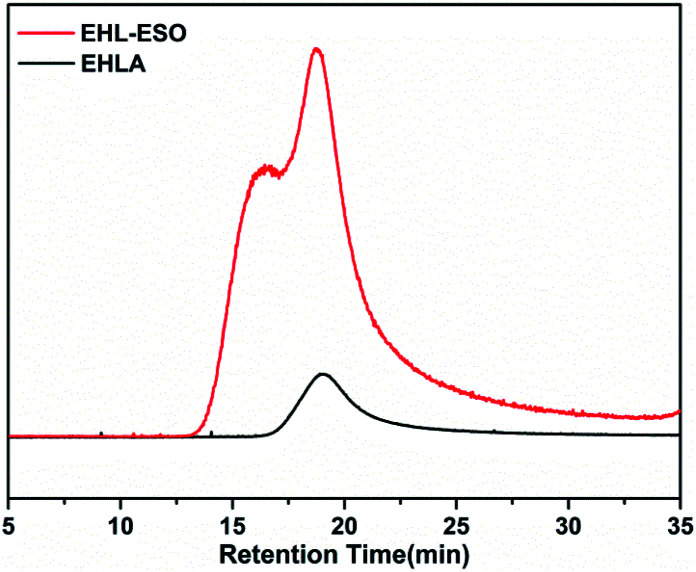
GPC spectra of EHLA and EHL-ESO.

Signals at 2.9–3.12 ppm (H8) was assigned to the epoxy ring (–CH and –CH_2_) of ESO.^29^ In the ^1^H NMR spectrum of EHL-ESO, some small peaks of H3 and H8 were retained and shifted to 8.33 ppm (H4) and 3.23 ppm (H7), respectively. The signal at 3.66 ppm (H6) was corresponded to the methoxy group,^30^ and a new signal at 3.43–3.52 ppm (H5) was attributed to the –OH protons.^31^ These results indicated that there was the occurrence of a ring-opening reaction between amino groups in EHLA and epoxy groups in ESO, corroborating the FTIR and ^1^H NMR analysis, which was further supported by GPC analysis (Fig. 3). EHLA demonstrated a peak molecular weight (*M*_p_) of 6376 g mol^−1^ and a weight-average molecular weight (*M*_w_) of 22 270 g mol^−1^. The *M*_p_ and *M*_w_ values of EHL-ESO were 36970 and 66160 g mol^−1^ (Table 2), respectively, indicating a substantial increase in mass from EHL-ESO.

**Table 2:** GPC data of EHLA and EHL-ESO

Sample	*M*_p_ (g mol^−1^)	*M*_w_ (g mol^−1^)	*M*_n_ (g mol^−1^)	Polydispersity index (*M*_w_/*M*_n_)
EHLA	6376	22 270	13 080	1.704
EHL-ESO	36 970	66 160	45 220	1.463

#### 3.1.2 Thermal properties

**Fig. 4:**
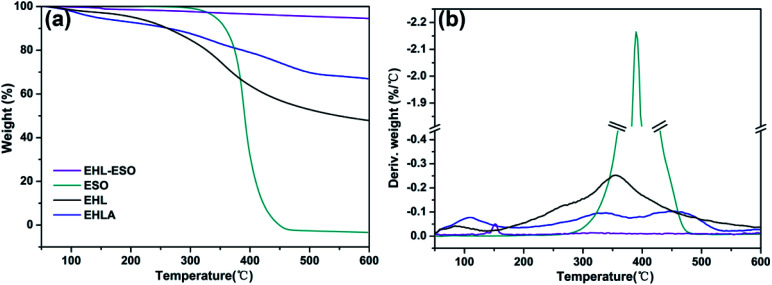
(a) TG and (b) DTG curves of EHL, EHLA, ESO, and EHL-ESO.

The thermal decomposition of EHL occurred in two stages (0–153 °C and 153–600 °C) (Fig. 4b), corresponding to the evaporation of residual water, and the cleavage of ether and carbon–carbon linkages, respectively.^26,32^ EHLA exhibited three stages of thermal decompositions (0–180 °C, 180–377 °C, and 377–600 °C) (Fig. 4b), belonging to the evaporation of residual moisture, lignin α-aryl ether and β-aryl ether bond cleavage accompanied by dehydration and decarboxylation, and C–C bond cleavage between lignin units and aliphatic side chains of aromatic rings, respectively.^33^ It can be seen from the TG curve (Fig. 4a) that the residual mass of ESO was 0% at 600 °C, which showed the lowest thermal stability. Moreover, the DTG curve (Fig. 4b) of ESO showed that there was only one stage of thermal decomposition (292–492 °C), indicating that all components of ESO decomposed completely in this temperature range.

After grafting ESO onto EHLA to form EHL-ESO, there was a stage of thermal decomposition, corresponding to residual moisture loss (Fig. 4b). More importantly, the residual masses of EHL, EHLA, and EHL-ESO were 47.9%, 66.97%, and 94.54%, respectively, after heating at 600 °C (Fig. 4a). These results confirmed that EHL-ESO was more thermally stable due to its crosslinked structure.

### 3.2 Characterization of soy protein-based adhesives

#### 3.2.1 FTIR analysis

**Fig. 5:**
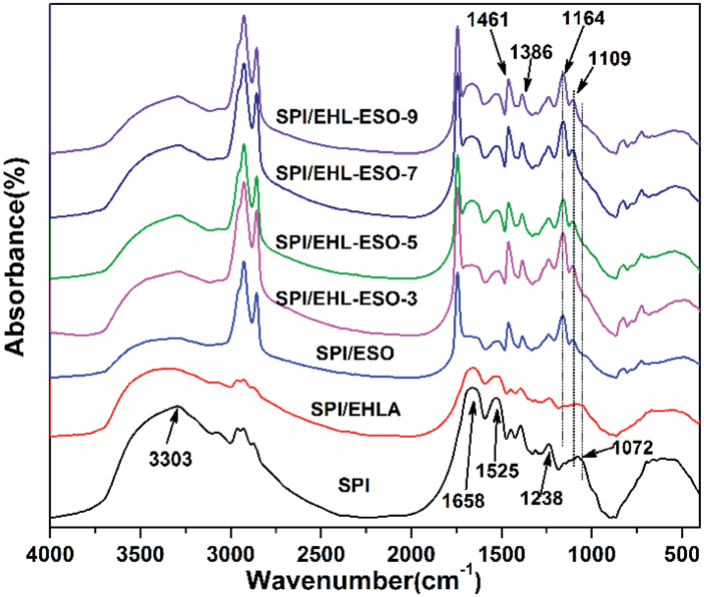
FTIR curves of different soy protein-based adhesives.

The interactions between the multifunctional crosslinker and soy protein molecules were investigated by FTIR analysis (Fig. 5). In the spectrum of SPI, the peaks of amide I, amide II, amide III, occurred at 1658, 1525, and 1238 cm^−1^, corresponding to C=O stretching, N–H bending, and N–H/C–N stretching vibration, respectively.^34,35^ The peak of free and bound NH– and O–H bending vibrations appeared at 3303 cm^−1^.

Introduction of EHLA reduced the absorption intensities of COO– (1386 cm^−1^), –OH or –NH (3303 cm^−1^), and C–O–C (1072 cm^−1^). The C=O stretching (1658 cm^−1^) of amide I also shifted to the lower wavenumber of 1650 cm^−1^. These results were possibly due to a hydrogen bonding interaction between EHLA and SPI.^17^ For SPI/ESO adhesive, new peaks appeared at 1741 (C=O in COOH or COOR), 827 (epoxy group), and 722 cm^−1^ ((CH_2_)*n*, HC=CH),^26^ the peak intensity of –CH vibrations belonging to CH_2_ and CH_3_ at 2929, 2854, and 1461 cm^−1^ increased,^21,24^ suggesting that ESO was successfully incorporated into the SPI matrix. Moreover, the peak intensity at 1525 cm^−1^ (N–H bending) decreased, demonstrating that the amino groups in SPI reacted with epoxy groups in ESO.

**Scheme 1:**
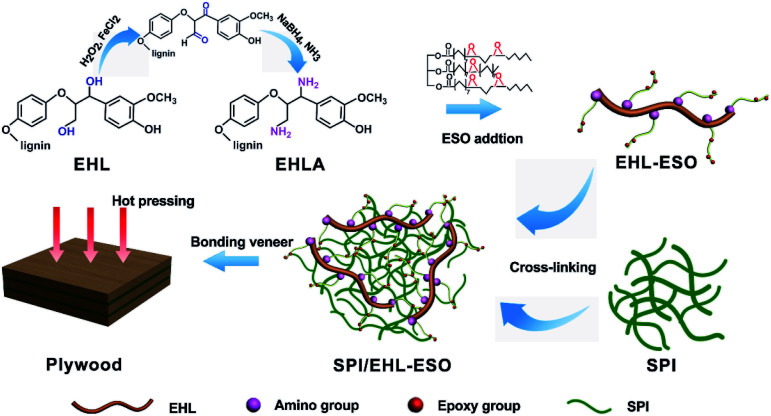
The crosslinking reactions process of SPI/EHL-ESO adhesives.

After incorporated EHL-ESO into the SPI, the broad bands at 1600 and 1508 cm^−1^ (aromatic C=C) of SPI/EHL-ESO disappeared, as they were overlapped by the strong C=O stretching of amide I and N–H bending of amide II vibration peaks of soy protein. In addition, the peak intensities at 2929, 2854, and 1461 cm^−1^ increased, the broad band at 1072 cm^−1^ (C–O–C) disappeared and a new peak attributed to saturated aliphatic ether at 1109 cm^−1^ occurred, implying that the crosslinker EHL-ESO was introduced.^47,49^ On the other hand, the intensity reduction of the peak at 3303 cm^−1^ (–OH and N–H) was contributed to a hydrogen bonding interaction between EHLA and SPI matrix. Moreover, the peaks at 1164 cm^−1^ (ester –CO) and 1741 cm^−1^ (C=O in COOH or COOR) appeared, and the absorption peak at 1386 cm^−1^ (COO–) was weakened, owing to an esterification reaction between epoxy groups in EHL-ESO and carbonyl groups/carboxyl group in SPI.^36,46–48^ At the same time, the peak intensity at 1525 cm^−1^ (N–H bending) decreased, implying that the amino groups in SPI reacted with epoxy groups. According to the above results, an interpenetrating crosslinked network structure was formed by the internal physiochemical reaction between EHL-ESO and soy protein molecules, which could increase the water resistance of soy protein-based adhesives (Scheme 1).

#### 3.2.2 Thermal properties

**Fig. 6:**
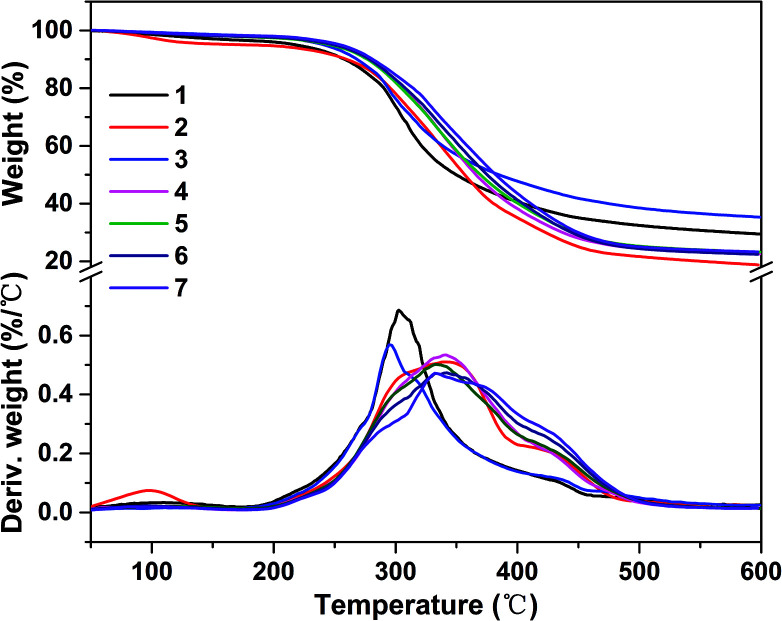
TG and DTG curves of different soy protein-based adhesives: (1) SPI, (2) SPI/ESO, (3) SPI/EHLA, (4) SPI/EHL-ESO-3, (5) SPI/EHL-ESO-5, (6) SPI/EHL-ESO-7, and (7) SPI/EHL-ESO-9.

The thermal properties result of soy protein adhesives are shown in Fig. 6. Pristine SPI and SPI/EHLA adhesives demonstrated two degradation processes (0–250 °C and 250–500 °C), corresponding to the degradation of small molecule and unstable chemical bonding, and the degradation of the main SPI molecular skeleton (Fig. 6), respectively.^37,39^ After introducing EHLA, the maximum degradation peak temperature (294 °C) was lower than that of pure SPI adhesive (302 °C), indicating a low crosslinking density between SPI and EHLA (Fig. 6). With the addition of ESO, the second degradation peak appeared as a shoulder at 423 °C, corresponding to the degradation of ESO.^38^ In addition, the maximum degradation peak temperature shifted to 340 °C, indicating an improvement in thermal stability, however, the adhesive demonstrated a severe mass loss.

After incorporating EHL-ESO modifier, SPI/EHL-ESO adhesives mainly presented three thermal degradation stages at approximately 0–250 °C, 250–400 °C, and above 400 °C (Fig. 6), which was corresponded to the degradation of small molecule and unstable chemical bonding, the degradation of the skeleton structure of soy protein, and the degradation of the lignin aromatic unit and the aliphatic components of the epoxy matrix, respectively.^37–39,42^

The maximum degradation temperature value (332–343 °C) of SPI/EHL-ESO adhesives was beyond higher than that of pristine SPI adhesive (302 °C), which can be likely due to the multiple interactions between EHL-ESO and the soy protein matrix.^39–42^ Moreover, the thermal stability of SPI/EHL-ESO adhesives was higher than that of pure UF resin and a “green” adhesive, composed of UF resin and cottonseed meal.^41^ In summary, EHL-ESO induced the formation of an interpenetrating crosslinked network structure, which enabled the formation of hydrogen bonds and other interactions that can increase the thermal stability, as confirmed by FTIR analysis.

#### 3.2.3 Adhesion strength in wood-based composite

**Fig. 7:**
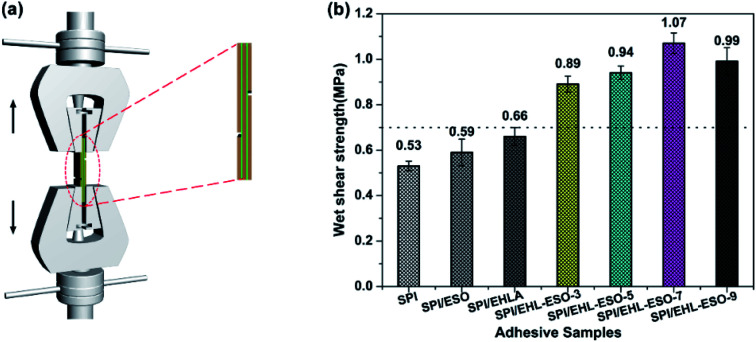
Wet shear strength of plywood bonded by different soy protein-based adhesives: (a) the schematic diagram of test instrument, (b) the results of wet shear strength.

The adhesion strength of the adhesive was measured according to China National Standards (GB/T 9846-2015). The schematic diagram of test instrument is shown in Fig. 7a, and the results of the wet shear strength are seen in Fig. 7b.

The wet shear strength values of SPI, SPI/ESO, and SPI/EHLA adhesives were 0.53, 0.59, and 0.66 MPa, respectively, which did not meet the requirements of interior-use plywood (≥0.7 MPa). The SPI and SPI/EHLA adhesives contained intermolecular hydrogen bonds that were easily disrupted by moisture, resulting in poor water resistance. The reaction occurred between epoxy groups in ESO and hydrophilic groups in SPI, but without a good compatibility,^20^ thus resulting in poor water resistance of SPI/ESO adhesive. The addition of EHL-ESO-3, EHL-ESO-5, EHL-ESO-7, or EHL-ESO-9 improved the wet shear strength of plywood bonded. A maximum value of 1.07 MPa was seen for SPI/EHL-ESO-7, which was a significant increase of 101.8% compared to that of pristine SPI adhesive. The main reason was that the epoxy groups in ESO can served as a “bridge” between SPI and EHLA *via* ring-opening reactions, strengthening the inherent molecular network.^21^ Moreover, the presence of multiple interactions between EHL-ESO and SPI can induce the formation of an interpenetrating and dense crosslinking network structure. This resulted in a significant decrease in cohesive failure of the adhesive bonds, and increase in the water resistance of SPI/EHL-ESO adhesive system.^43^ Increasing the amount of EHL-ESO, however, caused a slight decrease in the adhesive strength. The reason could be that the solution of excess EHL-ESO could produce the high viscosity adhesive, which could limit the ability of the adhesive to penetrate the wood substrate and establish mechanical interlocking.^44^

Table 3 presents the comparison of the adhesion strength, thermal stabilities, and sustainability of SPI/EHL-ESO adhesive with other bio-derived wood adhesives. For example, SPI/TGA/A-SSPS and SPI/CT_S_ bonded wood composites have wet shear strengths comparable to those bonded with SPI/EHL-ESO adhesive, but the thermal stabilities of these adhesives are worse than that of SPI/EHL-ESO adhesive. Moreover, the adhesion strength, thermal stabilities, and sustainability of SPI/EHL-ESO adhesive are superior to that of SPI/SSPS/SHMP, SM/LR, and SM/PHTO adhesives. Therefore, SPI/EHL-ESO adhesive has the potential to replace petroleum-based wood adhesives.

**Table 3:** Comparison of performance characteristics of SPI/EHL-ESO adhesive with other bio-derived wood adhesives

Adhesives	Wet shear strength (MPa)	Maximum degradation temperature (°C)	Sustainability	References
SPI/SSPS/SHMP	0.99	321	✓	(Yuan *et al.*, 2017)^40^
SPI/TGA/A-SSPS	1.07	322	✓	(Zhang *et al.*, 2018)^47^
SPI/CTs	1.07	300	✓	(Liu *et al.*, 2017)^5^
SM/LR	1.05	300	—	(Luo *et al.*, 2015)^17^
SM/PHTO	1.04	300	—	(Chen *et al.*, 2020)^50^
SPI/EHL-ESO	1.07	343	✓	Our study

#### 3.2.4 SEM analysis

**Fig. 8:**
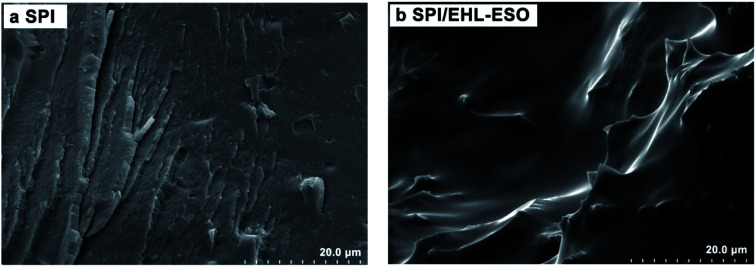
SEM images for cured adhesives of (a) the pristine SPI and (b) SPI/EHL-ESO.

Fig. 8 shows SEM of the cross-sectional morphology of the adhesives. It can be seen that pores and cracks were found in the pure SPI adhesive (Fig. 8a). This phenomenon indicated that moisture easily invaded these pores and cracks, causing swelling and destroying the adhesion. In addition, a loose and disordered surface morphology was observed, which indicated that an intertangling state was still remained in the protein molecules, resulting in a low water resistance.^45^ Compared with SPI adhesive microstructure, the fracture surface of SPI/EHL-ESO adhesive was smooth without pores as well as cracks, and the tortuous ridges were observed (Fig. 8b), implying a higher crosslinking density was formed by multiple interactions between the soy protein and EHL-ESO.^24,45^ Thus, the wet shear strength of SPI/EHL-ESO adhesives dramatically enhanced, verifying the analysis results of the adhesive strength.

## 4. Conclusions

In summary, a novel biopolymer (EHL-ESO) was successfully synthesized from both EHL and ESO and mixed with soy protein to produce a high-performance and bio-based soy protein adhesive. The results showed that modification of EHL could improve its reactivity and be as a multifunctional crosslinker. A dense crosslinking network structure was formed through the multiple interactions between epoxy groups (EHL-ESO) and hydrophilic groups (soy protein) during the hot-pressing process, which can enhance the thermal stability, water resistance and adhesion strength of soy protein adhesives. The maximum decomposition temperature of SPI/EHL-ESO adhesives (332–343 °C) was higher than that of pure SPI adhesive (302 °C). Moreover, the wet shear strength of SPI/EHL-ESO-7 adhesive reached a maximum value of 1.07 MPa, which was a 101.8% increase compared with the neat SPI adhesive. Therefore, SPI/EHL-ESO adhesives can be regarded as a novel, sustainable, and efficient bio-based wood adhesive with the potential to be a viable alternative to petroleum-derived adhesives.

## Abbreviations

SPI: Soy protein isolate
EHL: Enzymatic hydrolysis lignin
ESO: Epoxidized soybean oil
EHLA: Enzymatic hydrolysis lignin amine
EHL-ESO: A novel biopolymer
PF: Phenol formaldehyde
UF: Urea formaldehyde
PAE: Polyamidoamine-epichlorohydrin
THPTG: Trimethylolpropane triglycidyl ether
SL: Sorghum lignin
SHMP: Sodium hexametaphosphate
SSPS: Soybean soluble polysaccharide
TGA: Triglycidylamine
A-SSPS: Aminated SSPS
CT_S_: Condensed tannins
SM: Soybean meal
LR: Lignin-based resin
PHTO: Phenol hydroxymethylated tannin oligomer

## Conflicts of interest

The authors declare no conflicts of interest.

## Supplementary Material
